# The determinants of lung cancer after detecting a solitary pulmonary nodule are different in men and women, for both chest radiograph and CT

**DOI:** 10.1371/journal.pone.0221134

**Published:** 2019-09-11

**Authors:** Elisa Chilet-Rosell, Lucy A. Parker, Ildefonso Hernández-Aguado, María Pastor-Valero, José Vilar, Isabel González-Álvarez, José María Salinas-Serrano, Fermina Lorente-Fernández, M. Luisa Domingo, Blanca Lumbreras

**Affiliations:** 1 Public Health, History of Science and Gynaecology Department, Miguel Hernández University, Alicante, Spain; 2 CIBER en Epidemiologia y Salud Pública, Madrid, Spain; 3 Radiodiagnostic Department, Peset Hospital, Valencia, Spain; 4 Radiodiagnostic Department, San Juan Hospital, San Juan de Alicante, Spain; 5 Computer Department, San Juan Hospital, San Juan de Alicante, Spain; Mount Sinai Health System, UNITED STATES

## Abstract

**Objectives:**

To determine the factors associated with lung cancer diagnosis and mortality after detecting a solitary pulmonary nodule (SPN) in routine clinical practice, in men and in women for both chest radiograph and CT.

**Materials and methods:**

A 5-year follow-up of a retrospective cohort of of 25,422 (12,594 men, 12,827 women) patients aged ≥35 years referred for chest radiograph or CT in two hospitals in Spain (2010–2011). SPN were detected in 893 (546 men, 347 women) patients. We estimated the cumulative incidence of lung cancer at 5-years, the association of patient and nodule characteristics with SPN malignancy using Poisson logistic regression, stratifying by sex and type of imaging test. We calculated lung cancer specific mortality rate by sex and SPN detection and hazard rates by cox regression.

**Results:**

133 (14.9%) out of 893 patients with an SPN and 505 (2.06%) of the 24,529 patients without SPN were diagnosed with lung cancer. Median diameter of SPN in women who developed cancer was larger than in men. Men who had a chest radiograph were more likely to develop a lung cancer if the nodule was in the upper-lobes, which was not the case for women. In patients with an SPN, smoking increased the risk of lung cancer among men (chest radiograph: RR = 11.3, 95%CI 1.5–83.3; CT: RR = 7.5, 95%CI 2.2, 26.0) but smoking was not significantly associated with lung cancer diagnosis or mortality among women with an SPN. The relative risk of lung cancer diagnosis in women with SPN versus those without was much higher compared to men (13.7; 95%CI 9.2, 20.4 versus 6.2; 95%CI 4.9,7.9).

**Conclusion:**

The factors associated with SPN malignancy and 5-year lung cancer mortality were different among men and women, especially regarding smoking history and SPN characteristics, where we observed a relatively high rate of lung cancer diagnosis among female non-smokers.

## 1. Introduction

Solitary pulmonary nodules (SPN) are frequently detected during interpretation of imaging tests in clinical routine practice, and their presence raises the suspicion of lung cancer. Although several guidelines provide recommendations in SPN management [1–3], and some efforts have been made to validate them in general population[1], the guidelines are mainly based on data from screening studies using CT.

Moreover, the scientific literature shows a differential risk of lung cancer in women and men[4–8]. The main explanation for this difference is related to changing patterns of smoking and tobacco habit for men and women [9]. However, epidemiological studies have shown conflicting results, some presenting a higher risk of lung cancer among women who smoke compared to men, while other studies found either no differences or a higher risk for men [10]. Furthermore, lung cancer among never-smokers is more common in women than in men, probably due to a greater incidence of passive smoking between women [4, 6–8], as well as, different carcinogenic pathways [11]. Therefore, and given that the susceptibility to smoking-related lung cancer may differ between men and women [12], extrapolating risk estimates for lung cancer in men to women could underestimate the adverse impact of smoking in women. Women also show a better survival rate [13], suggesting that the natural history of lung cancer may differ in women and men.

The Fleischner Society published guidelines in 2005 to deal with the management of incidentally detected pulmonary nodules in CT, and in 2013 for subsolid nodules [14]. These guidelines were updated in 2017 [3] and they are widely established [15]. Moreover, the American College of Chest Physicians developed two guidelines, the last updated in 2013 [2]. These recommendations do not include sex as predictor of lung cancer risk. The British Thoracic Society (BTS) updated the available evidence in 2015 and published different recommendations according to the nodule route of detection for CT, which have been recently validated in routine practice [1]. The BTS recommends the use of mathematical prediction models, such as the Herder and the Brock model, and includes sex as a clinical factor. In the United Kingdom, these guidelines have superseded the Fleischner recommendations [16].

These recommendations include patients with SNP detected for CT, because they draw from the evidence in lung cancer screening trials. Nevertheless, in clinical practice, SPN are usually detected by both chest radiograph and CT. Given the difference in nodule characteristics and variables associated with nodule prevalence for patients according to the imaging test, it is important to study these techniques separately in general population [17]. To clarify any sex difference in lung cancer risk which should be incorporated into the available recommendations, it is necessary to evaluate a population cohort showing all the pathways of nodule detection in routine practice.

We previously showed differences in lung cancer risk between SPN detected by chest radiograph (8.3%) and those detected by CT (12.4%) within 18 months of detection [17, 18]. However, the 18-month follow-up period could have underestimated the risk of cancer because some lesions are slow growing. This paper presents the different risk for men and women during the 5-year follow up of our retrospective cohort of SPN detected by chest radiograph or CT during routine clinical practice.

The aim of the study is to determine the factors associated with lung cancer diagnosis and mortality after detecting SPN for both chest radiograph and CT in routine clinical practice, in both men and in women during the 5-year follow up of the patients.

## 2. Material and methods

Institutional Review Board approval (University Miguel Hernandez Committee Ref DSP-BLL-001-10) was obtained.

### 2.1 Patients

We analysed a retrospective cohort study of patients aged ≥35 years (because lung cancer is rare in those under 35 years [19]) referred for thoracic imaging for non-screening reasons (such as preoperative evaluation) to two hospitals in the Valencian Community (Spain) from within the hospital and from primary health care centers during in 2010 and 2011 [17, 18]. Both hospitals belong to the National Health Care System and are referral hospitals for all individuals living in their respective geographical catchment area. Lung cancer screening is not implemented in this area. We classified the patients according to the imaging test where the nodule was firstly detected: a) Patients, who first had a chest radiograph and subsequently had a CT, were categorized as having had a chest radiograph, and b) Patients who first had a CT were categorized as CT. Patients previously diagnosed with lung cancer and patients who were not resident in the Valencian Community were excluded. We present here a 5 year-follow-up of the cohort with 25,422 patients (12,651 men and 12827 women), mean age 64.5±14.9 years (64.9±15.3 years in women and 64.1±14.5 years in men).

### 2.2 Data collection

#### 2.2.1 Detection and description of the SPN

Eight expert radiologists (all of them with more than 10 years of experience) determined the presence of SPN in thoracic studies of the patients initially included. We limited our study to nodules between 3 and 30 millimeters of size. Intrapulmonary lymph nodes and pseudolesions, when detected, were excluded from our study. Given the different sensitivity among radiography and CT, we present the results separately.

Chest radiographs were obtained with the standard technique in digital format (CR Philips). CT imaging tests were obtained with slice thicknesses of 3mm or less (2 mm, 1.5 and 1.25) according to the different clinical situations and the equipment used, 120 KvP and variable mAs. The nodules were measured using calipers in the PACS workstations in their largest diameter in the posterior anterior and lateral radiograph. CT lung window settings (1550/-600) were used to measure nodule size in the largest diameter. Mediastinal window settings (350/50) were also used to further detect calcification or fat (<40 Hounsfield units) within the nodule.

The radiologists described nodule characteristics: a) size, in mms, and also expressed as mean (sd) in diameter; b) nodule shape, smooth or irregular (lobular or spiculated); c) location, and d) for those patients who underwent a CT, nodule consistency (solid, partly solid, ground glass, calcification or not specified). The first 300 tests included were evaluated independently by the 8 radiologists and we previously evaluated inter-observer agreement in aspects such as nodule size, shape or consistency [17]. We also evaluated intra-observer agreement of these characteristics.

#### 2.2.2 Patients’ characteristics

Selected clinical and demographic variables were extracted from the medical records for all patients: type of test performed (CT or radiograph); care setting (inpatient or outpatient); reason for test (respiratory, non-respiratory, preoperative, extrapulmonary neoplasm) and patient characteristics (age, sex).

In the 893 patients with SPN, we collected extra information from the medical records: smoking habit (non-smokers, current or former smokers), previous malignancy and presence of COPD.

Patients’ data were completely anonymised before access, therefore researchers had no access to potentially identifying information.

### 2.3 Follow-up

All participants were followed up for 5 years from nodule detection. To determine lung cancer frequency and all-cause mortality among the cohort, we linked our database with the electronical medical record registers from both hospitals. In those patients where a confirmed diagnosis or suspicion of lung or thoracic cancer appeared in the medical records, we confirmed the exact date of lung cancer diagnosis, and specific cause of death against histopathological records and the Hospital Minimum Basic Data Set (MBDS), which registers all clinical interventions performed and diagnoses in patients who have been admitted to the hospital.

The lung cancer diagnosis was made according to established clinical guidelines [20], by histopathological examination of resection specimens or cytopathological examination of needle-aspiration biopsy samples.

### 2.4 Statistical analysis

All data were computerised anonymously and checked to discard errors. Statistical precision was determined through the calculation of 95% confidence intervals using the appropriate method according to the type of measurement and the available data. All analyses carried out with the statistical programme Stata/SE 12.1 (Stata Corp., College Station, Texas, USA).

We estimated the prevalence of SPN, and the cumulative incidence of lung cancer at 5 years according to patient and nodule characteristics for men and for women separately. We calculated age adjusted relative risk of lung cancer in women and men. In patients with SPN, we estimated the relative risk of lung cancer at 5 years of follow-up associated to sociodemographic and clinical variables, and nodule characteristics using Poisson logistic regression (the model included only predictors that reached statistical significance p<0.005). Analyses were stratified by type of imaging test, as has been previously acknowledged, characteristics of patients that undergo a chest radiograph or CT differ [17]. We calculated lung cancer specific mortality rate by sex and SPN detection and performed survival analysis using Cox proportional hazards modelling.

## 3. Results

**Fig 1:**
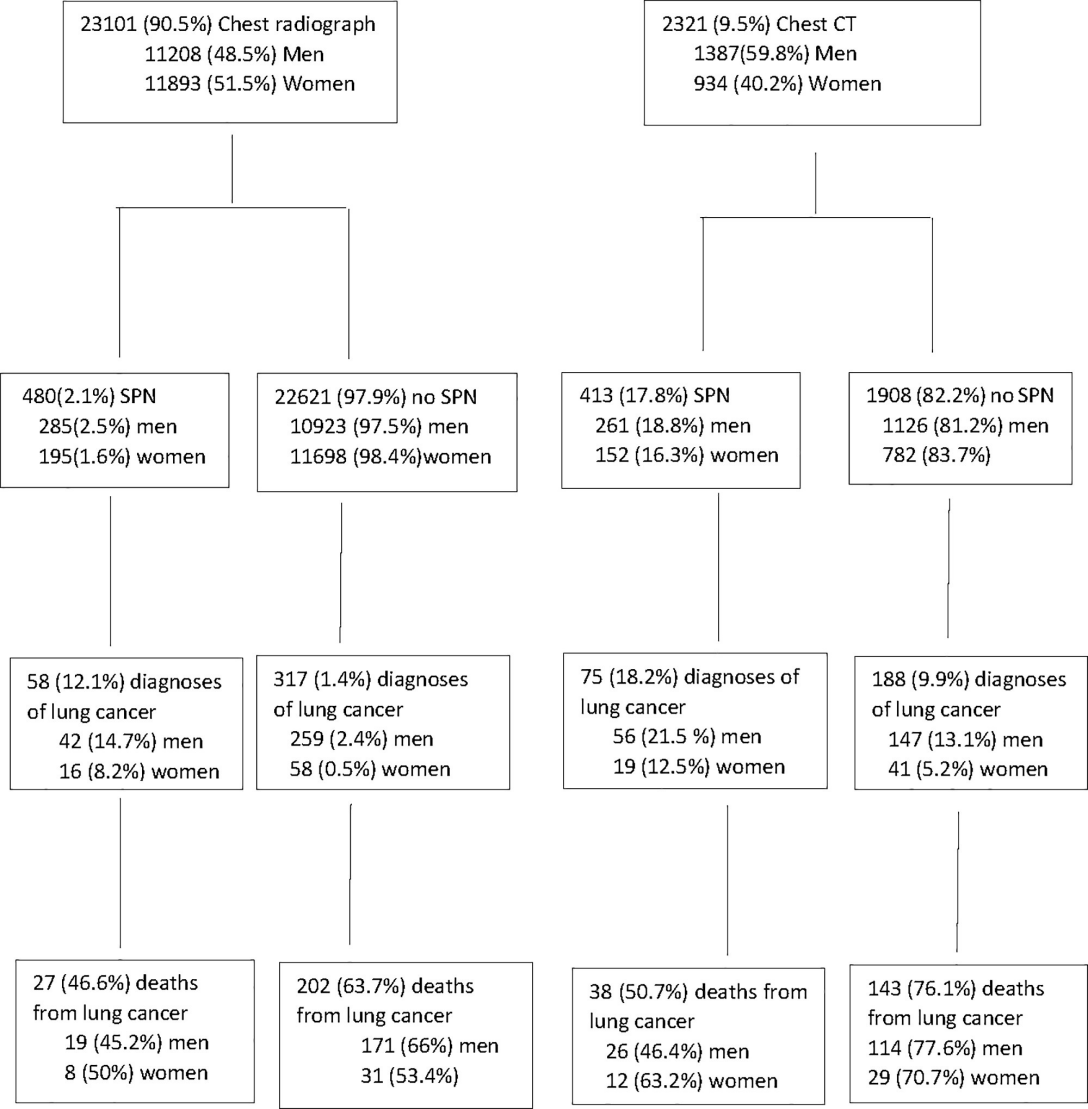
Flow diagram showing the 5 year follow up period of the 25,422 patients undergoing thoracic imaging tests during routing clinical practice.

Table 1 shows baseline data of the patients who underwent a chest radiograph or CT for non-screening reasons. For women and men, patients who underwent a CT were older and more frequently were outpatients, former or current smokers, underwent a imaging test due to respiratory symptoms and had COPD diagnosis. Of the 25,422 evaluated patients, 23,101 (90.5%) underwent a chest radiograph as first imaging test (48.5% men) and 2,321 (9.5%) patients underwent a CT (59.8% men) (Fig 1). 139 (15.6%) out of 893 patients with an SPN detected and 505 (2.06%) out of the 24,529 patients without SPN developed lung cancer during the follow-up period. Lung cancer rate was 3.719/1000 person-years (95%CI 3.403, 4.035) for patients without an SPN and 26.455/1000 person-years (95%CI 22.169, 31.569) in patients with an SPN.

**Table 1:** Baseline demographics data of men and women who underwent a chest radiograph or CT for non-screening reasons in two hospitals during 2010 and 2011.

	Men			Women		
	Chest Radiograph (11208)	CT (1387)	Total (12595)	Chest Radiograph (11893)	CT (934)	Total (12827)
**Age (years)**						
<50	2485 (22.2)	183 (13.2)	2668 (21.2)	2564 (21.6)	167 (17.9)	2731 (21.3)
50–59	1911 (17)	279 (20.1)	2190 (17.4)	2049 (17.2)	190 (20.3)	2239 (17.5)
60–69	2599 (23.2)	342 (24.7)	2941 (23.3)	2445 (20.6)	226 (24.2)	2671 (20.8)
>70	4206 (37.5)	582 (42)	4788 (38)	4833 (40.6)	351 (37.6)	5184 (40.4)
Not specified	7 (0.1)	1 (0)	8 (0.1)	2 (0)	0 (0)	2 (0)
**Setting**						
Inpatient	1746 (15.6)	383 (27.7)	2129 (16.9)	1569 (13.2)	180 (19.3)	1749 (13.6)
Outpatient	9453 (84.4)	1001 (72.3)	10454 (83.1)	10319 (86.8)	754 (80.7)	11073 (86.4)
Not specified	9 (0.1)	3 (0.2)	12 (0.1)	5 (0)	0 (0)	5 (0)
**Reason for requesting imaging test**						
Respiratory	2103 (18.8)	524 (37.8)	2627 (20.9)	1884 (15.8)	282 (30.2)	2166 (16.9)
Non- respiratory	3107 (27.7)	262 (18.9)	3369 (26.8)	3461 (29.1)	203(21.7)	3664 (28.6)
Extrapulmonary neoplasm	543 (4.8)	198 (14.3)	741 (5.9)	542 (4.6)	152 (16.3)	694 (5.4)
Preoperative	2179 (19.4)	32 (2.3)	2211 (17.5)	2614 (22)	24 (2.6)	2638 (20.6)
Not specified	3276 (23.2)	371 (26.8)	3647 (28.9)	3392 (28.5)	273 (29.2)	3665 (28.6)
**Smoking habit**						
Never	59 (0.5)	51 (3.7)	110 (0.9)	40 (0.3)	41 (4.4)	81 (0.6)
Former and current	178 (1.6)	166 (12)	344 (2.7)	97 (0.8)	86 (9.2)	183 (1.4)
Not specified	10971 (97.9)	1170 (84.4)	12141 (96.4)	11756 (98.9)	807 (86.4)	12563 (97.4)
**COPD**						
No	197 (1.8)	164 (11.8)	361 (66.2)	161 (83)	128 (84.2)	289 (83.5)
Yes	88 (0.7)	96 (6.9)	184 (33.8)	33 (17)	24 (15.8)	57 (16.5)
Not specified	10923 (97.5)	1127 (81.3)	1127 (81.3)	11699 (98.4)	782 (83.7)	12481 (97.3)

Women with an SPN had a lung cancer rate of 17.674/1000 person-years (95%CI 12.564, 24.860) which was higher than the lung cancer rate in women without SPN (1.380/1000 person-years; (95%CI 1.127, 1.689). Age-adjusted relative risk of lung cancer in women with SPN versus those without was 13.7 (95%CI 9.2, 20.4). In men, the lung cancer rate was 32.348/1000 person-years (95%CI 26.310, 39.772) in those patients with SPN and 6.22/1000 person-years (95%CI 5.637, 6.8651) in patients without SPN. Age-adjusted relative risk of lung cancer in men with SPN versus those without was 6.2 (95%CI 4.9, 7.9).

After adjusting for those patient and SPN characteristics, the rate of lung cancer diagnosis was similar in men and women (HR = 1.13 (95%CI 0.69, 1.84), while in patients without an SPN it was higher in men (HR = 4.17 (95%CI 3.28–5.31).

### 3.1 Mortality rates in men and women with lung cancer diagnosis

Of the 113 patients with SPN detected by either CT or radiography and diagnosed with lung cancer, 65 (48.9%), and of the 505 patients without SPN and diagnosed with lung cancer, 345(68.2) died within 5 years. The mortality rate in patients with lung cancer diagnosis varied according to previous diagnosis of SPN. Those patients without an SPN had lower mortality (2.55/1000 person-years; 95%CI 2.291, 2.837) than those with an SPN (13.335/1000 person-years; 95%CI, 10.396, 17.104). Mortality in women with an SPN (10.711/1000 person-years; (95%CI, 6.91, 16602) was higher than the mortality observed in women without an SPN (0.851/1000 person-years; 95%CI 0.658, 1.101). In men, mortality rate was 15.096 persons-years (95%CI 12.903, 22.720) in patients with SPN and 4.37 (95%CI 3.882 4.912) in those without.

In patients with an SPN and lung cancer diagnosis, men who were current or former smokers were more likely to die within 5 years (Hazard Ratio (HR) 7.3, 95%CI 1.8, 30.1); however current or former smoking was not significantly associated with mortality among women (HR 0.6, 95%CI 0.2, 1.7).

### 3.2 Solitary pulmonary nodules detected by chest radiograph

Of the 23,101 patients who underwent a chest radiograph, 480 (2.1%) patients had an SPN, 195 (1.6%) women compared to 285 (2.5%) men. Of these, 58 (12.1%) patients were diagnosed with lung cancer, 16 (8.2%) women compared to 42 (14.7%) men, and 27 (46.6%) died, 8 (50%) women compared to 19 (45.2%) men during the follow-up (Fig 1). Of the 22,621 (97.5%) patients without an SPN, 317 (1.4%) developed lung cancer later during the follow-up: 58 (0.5%) women and 259 (2.4%) men), and 202 (63.7%) died of lung cancer within 5 years (31(53.4%) women and 171 (66%) men) (Fig 1).

Considering all patients that had an SPN, the risk of lung cancer was higher in older patients, in former or current smokers, and in those with a diagnosis of COPD (Table 2). Comparing women and men, we show lung cancer diagnosis was significantly more frequent in male smokers (21.4%) than in female smokers (10.3%, p = 0.021). However, lung cancer was more frequent in women who had never smoked (12.5%) than in men who had never smoke (1.7%, p = 0.027). In Table 3, we show that among men, former or current smoking (RR = 11.3, 95%CI 1.5, 83.3), and diagnosis of COPD (RR = 1.7, 95%CI 1.1, 3.2) significantly increased the risk of lung cancer. Smoking habit and diagnosis of COPD were not a significant risk for women (RR = 0.8, 95% 0.2, 2.5 and RR 1.6, 95%CI 0.4–6.6, respectively). Oupatient women were less likely to develop lung cancer than inpatients (RR = 0.2, 95%CI 0.3–0.9).

**Table 2:** Frequency of lung cancer within 5 years of detecting an SPN by chest radiograph or CT and its distribution according to patient’s characteristics.

	Chest Radiograph					CT				
	Total 480	Cancer, n (%) 58(12.1)	p.value	aRR (CI)	p. value	Total 413	Cancer, n (%) 75 (18.2)	p. value	aRR (CI)	p. value
**Age (years)**										
<50	76	3 (3.9)	0.077	1	0.058	50	7 (14)	0.868		
50–59	85	13 (15.3)		3.4 (0.9–12.3)	0.048	87	16(18.4)		1.1 (0.4–2.6)	0.897
60–69	133	18 (13.5)		3.5 (1–12.1)	0.09	126	25 (19.8)		1.3 (0.5–2.9)	0.619
≥70	186	24 (12.9)		2.9 (0.9–12.6)		150	27 (18)		1.1 (0.5–2.5)	0.851
**Setting**										
Inpatient	51	10 (19.6)	0.081	1		86	24 (27.9)	0.008	1	
Outpatient	429	48 (11.2)		0.7 (0.3–1.4)	0.394	327	51 (15.6)		0.6 (0.3–0.9)	0.021
**Reason for requesting imaging test**										
Respiratory	86	16 (18.6)	0.225	1		79	14 (17.7)	0.619	1	
Non-respiratory	135	15 (11.1)		0.7 (0.3–1.4)	0.325	86	13 (15.1)		0.8 (0.4–1.8)	0.590
Extrapulmonary neoplasm	102	13 (12.8)		1.1 (0.5–2.3)	0.892	108	17 (15.7)		0.9 (0.5–1.9)	0.892
Preoperative	50	3 (6)		0.4 (0.1–1.4)	0.150	51	14 (27.5)		1.6 (0.7–3.3)	0.245
Not available	107	11 (10.3)		0.6 (0.3–1.5)	0.363	89	17 (19.1)		1.1 (0.5–2.4)	0.744
**Smoking habit**										
Never	99	6 (6.1)	<0.001	1		92	9 (9.8)	<0.001	1	
Former and current	275	48 (17.5)		2.6(1.1–6.1)	0.029	252	60 (23.8)		2.2 (1–4.8)	0.030
Not specified	106	4 (3.8)		0.5 (0.1–1.8)	0.269	69	6 (8.7)		0.8 (0.2–2.4)	0.730
**Previous malignancy**										
No	313	30 (9.6)	0.066	1		286	48 (16.7)	0.260	1	
Yes	167	28 (16.8)		1.7 (0.9–2.8)	0.062	126	27 (21.4)		1.3 (0.8–2.2)	0.250
COPD*										
No	358	33 (9.2)	0.002	1		292	46 (15.8)	0.101	1	
Yes	121	24 (19.8)		1.8(1–3.1)	0.036	120	28 (23.3)		1.4 (0.8–2.2)	0.069

aRR: adjusted relative risk

*two missing data (one patient in chest radiograph group and one patient in CT group)

**Table 3:** Frequency of lung cancer within 5 years of detecting an SPN by chest radiograph stratified by sex, and its distribution according to patient’s characteristics.

	Male					Female				
	Total 285	Cancer, n (%) 44 (15.4)	p.value	aRR (CI)	p.value	Total 195	Cancer, n (%) 18 (9.2)	p.value	aRR (CI)	p.value
**Age (years)**										
<50	39	2 (5.1)	0.193	1		37	1 (2.7)	0.325	1	
50–59	47	10 (21.3)		3.3 (0.7–15.8)	0.136	38	3 (7.9)		3.1 (0.3–30.4)	0.333
60–69	81	11 (13.6)		2.2 (0.5–10.6)	0.307	52	7 (13.5)		6.8 (0.8–57.8)	0.079
≥70	118	19 (16.1)		2.3 (0.5–10.3)	0.287	68	5 (7.4)		2.8 (0.3–26.2)	0.368
**Setting**										
Inpatient	37	7 (18.9)	0.442	1		14	3 (21.4)	0.061	1	
Outpatient	248	35 (14.1)		0.9 (0.4–2)	0.762	181	13 (7.2)		0.2 (0.3–0.9)	0.043
**Reason for requesting imaging test**										
Respiratory	50	12 (24)	0.273	1		36	4 (11.1)	0.247	1	
Non-respiratory	74	11 (14.9)		0.7 (0.3–1.6)	0.392	61	4 (6.6)		1.2 (0.2–7.2)	0.827
Extrapulmonary neoplasm	63	7 (11.1)		0.7 (0.3–1.8)	0.458	39	6 (15.4)		14.5 (0.8–28.1)	0.111
Preoperative	35	3 (8.6)		0.6 (0.2–2.1)	0.383	15	0 (0)		0 (-)	0.997
Not available	63	9 (14.3)		0.8 (0.3–1.9)	0.556	44	2 (4.5)		1.2 (0.1–9.7)	0.879
**Smoking habit**										
Never	59	1 (1.7)	<0.001	1		40	5 (12.5)	0.091	1	
Former and current	178	38 (21.4)		11.3(1.5–83.3)	0.017	97	10 (10.3)		0.8 (0.2–2.5)	0.641
Not specified	48	3 (6.2)		3.9 (0.4–37.6)	0.243	58	1 (1.7)		——	—
**Previous malignancy**										0.299
No	174	20 (11.5)	0.194	1		139	10 (7.2)	0.418	1	
Yes	111	21(19.8)		1.7 (0.8–3.4)	0.102	56	6 (10.7)		1.9 (0.6–6)	
COPD*										
No	197	22 (11.2)	0.003	1		161	11 (6.8)	0.300	1	0.487
Yes	88	20 (22.7)		1.7 (1.1–3.2)	<0.001	33	4 (12.1)		1.6 (0.4–6.6)	

aRR: adjusted relative risk

*One female patient missing data

The age adjusted relative risk of developing lung cancer in patients with an SPN versus patients without an SPN was higher in women (RR = 15.9, 95%CI 9.2, 27.8) than in men (RR = 5.7, 95%CI 4.1, 7.9).

Regarding nodule characteristics, SPN diameter was larger in patients who developed lung cancer (median 18.8mm ±7.3) compared to those who did not (median 10.5mm ±6.0) (p<0.001). 15.2% of patients with an SPN located in the upper lobe developed lung cancer within 5 years, as did 48.0% of those with a spiculated border. 60% of women and 45% of men with SPN with spiculated borders developed lung cancer within 5 years. There were differences in lung cancer risk according to the SPN location in men (p = 0.037) but this was not showed in women (p = 0.557) (S1 Table). No patient with an SPN of 3-4mm developed lung cancer. However, lung cancer risk increased as nodules size increased, particularly in SPN larger than 12mm (lung cancer risk: 18.6%), and reached the highest risk (46.2%) in those patients with SPN bigger than 28mm (S2 Table).

### 3.3 Solitary pulmonary nodules detected by CT

Of the 2,321 (9.5%) patients who underwent a CT, 413 (17.8%) had SPN detected (152 (16.3%) women compared to 261 (18.8%) men). Of these, 75 (18.2%) were diagnosed with lung cancer (19 (12.5%) women compared to 56 (21.5%) men), and 38 (50.7%) died within 5 years, (12 (63.2%) women 26 (46.4%) men). Of the 1908 (82.2%) patients without SPN, 782 (83.7%) women and 1126 (81.2%) men, 188 (9.9%) developed lung cancer later in the follow-up (41 (5.2%) women and 147 (13.1%) men) and 143 (76.1%) died from lung cancer within 5 years, (29 (70.7%) women and 114 (77.6%) men) (Fig 1).

Among all patients with an SPN detected by CT, risk of lung cancer was higher in former or current smokers (RR = 2.2, 95%CI 1, 4.8) (Table 1). Comparing women and men, we show lung cancer diagnosis was significantly more frequent in male smokers (30.1%) than in female smokers (11.6%, p<0.001) and men with a diagnosis of COPD. On the contrary, lung cancer was more frequent in women (11.6%) who had never smoked than men who had never smoked (5.9, p<0.001). When stratifying by sex (Table 4), men who were former or current smokers had a higher risk of lung cancer than those who had never smoked (RR = 7.5, 95%CI 2.2, 26.0), but this pattern was not observed among women (RR = 0.7, 95%CI 0.3, 2.1). Women with COPD were not more likely to develop into lung cancer compared to women without COPD (RR = 2.6, 95%CI 0.2–7.6), the same was not observed among men (RR = 0.9, 95%CI 0.5, 1.8). Outpatient men were less likely to develop into lung cancer than inpatients (RR = 0.5, 95%CI 0.3–0.9).

**Table 4:** Frequency of lung cancer within 5 years of detecting an SPN by CT stratified by sex, and its distribution according to patient’s characteristics.

	Male					Female				
	Total 261	Cancer, n (%) 59 (22.6)	p.value	aRR (CI)	p.value	Total 480	Cancer, n (%) 58(12.1)	p.value	aRR (CI)	p.value
**Age (years)**										
<50	39	5 (17.9)	0.843	1		22(100)	2 (9.1)	0.891	1	
50–59	47	10 (20.9)		0.9 (0.3–2.8)	0.892	39(100)	6 (15.4)		1.3 (0.3–7.2)	0.729
60–69	81	20 (24.7)		1.1 (0.4–3.1)	0.813	45(100)	5 (11.1)		1.2 (0.2–6.8)	0.818
≥70	118	21 (20.2)		1 (0.4–2.7)	0.986	46 (100)	6 (13)		1.2 (0.3–6.4)	0.822
**Setting**										
Inpatient	37	21 (33.9)	0.006	1		24(100)	3 (12.5)	0.100	1	
Outpatient	248	35 (17.6).9)		0.5 (0.3–0.9)	0.025	128(100)	16 (12.5)		0.9 (0.2–3.2)	0.831
**Reason for requesting imaging test**										
Respiratory	50	12 (20.3)	0.808	1		20 (100)	2 (10)	0.109	1	
Non-respiratory	74	12 (23.5)		0.9(0.4–2.2)	0.957	35(100)	1 (2.9)		0.4 (0.3–4.1)	0.405
Extrapulmonary neoplasm	63	13 (19.1)		0.9 (0.4–2)	0.810	40(100)	4 (10)		3.1 (0.2–7.3)	0.811
Preoperative	35	9 (29)		1.3 (0.5–3.1)	0.584	20(100)	5 (25)		3.1 (0.6–17.3)	0.192
Not available	63	10 (19.2)		0.8 (0.2–1.9)	0.651	37(100)	7 (18.9)		2.5 (0.4–11)	0.358
**Smoking habit**										
Never	59	3(5.9)	<0.001	1		41(100)	6 (14.6)	0.889	1	
Former and current	178	50 (30.1)		7.5 (2.2–26.0)	0.008	86(100)	10 (11.6)		0.7 (0.3–2.1)	0.576
Not specified	48	3 (6.8)		1.2 (0.2–6.4)	0.863	25(100)	3 (11.6)		0.8 (0.2–3.2)	0.717
**Previous malignancy**										0.733
No	174	35 (19.2)	0.312	1		104(100)	13 (12.5)	0.964	1	
Yes	111	21 (26.6)		1.5 (0.8–3)	0.211	47 (100)	6 (12.8)		1.2 (0.4–3.3)	
COPD*										
No	197	33 (20.1)	0.857	1		128(100)	13 (10.2)	0.044	1	0.081
Yes	88	22 (22.9)		0.9 (0.5–1.8)	0.886	24(100)	6 (25)		2.6 (0.2–7.6)	

aRR: adjusted relative risk

*One male patient missing data

The age adjusted relative risk of developing lung cancer in patients with an SPN versus those without an SPN was higher for women (RR = 2.3, 95%CI 1.3–3.9) than in men (RR = 1.6, 95%CI 1.2, 2.2).

The SPN diameter was larger in patients who developed lung cancer (17mm ± 7.7) compared to those who did not (8.9mm±5.6) (p<0.001). 47.8% of those patients with an SPN of spiculated border (95% CI 35.7, 59.8) developed lung cancer. Disaggregated data shows that median diameter of SPN in women who developed cancer was 12.7mm (±6.38) and 19.1mm in men (±7.57) (S3 and S4 Tables).

## 4. Discussion

The factors associated with SPN malignancy and 5-year lung cancer mortality were different among men and women, especially regarding smoking history -where we observed a relatively high rate of lung cancer diagnosis among women classified as non-smokers-. Even though cumulative incidence of lung cancer is higher in males, the adjusted lung cancer rate ratio for men versus women was not statistically significant in patients with SPN. Moreover, our study shows that in a clinical-based population, the presence of SPN detected by chest radiograph or CT is a better predictor of lung cancer diagnosis in women.

Size and spiculated border were associated with SPN malignancy in men and women, as in former studies [2, 3]. In our population, outpatients were less likely to develop lung cancer, maybe this fact was related to the presence of less comorbidities in outpatients than in inpatients.However, some factors associated with SPN malignancy were different in men and in women. For instance, median diameter of SPN in women who developed cancer was larger than in men. In addition, in patients who had a chest radiograph, men were more likely to develop a lung cancer if the nodule was in the upper-lobes, which was not the case for women. A recent study has developed eight mathematical models to support clinicians in their decision-making [21]. These models include some predictors of lung cancer like upper lobe location, but they neither include sex nor the type of imaging test performed. Previous studies have shown sex- and histologic type- differences in the association of smoking with lung cancer risk [12, 22].Thus, it is essential to study further the possible causes underlying the interaction sex-lung nodule characteristics.

Patient characteristics, such as advanced age and history of smoking, have been associated with a higher risk of malignant SPN and consequently are included in all available guidelines. However, former and current smoking was only a risk factor for lung cancer and specific mortality in men who underwent either a chest radiograph or a CT. The lack of relative increase in risk among current or former smoking women can be explained by the high rates of lung cancer diagnosis among women who have never smoked [8]. Passive smoking and environmental smoke are known to have some influence [11], and oestrogen may also influence the development and progression of lung cancer in peri-menopausal and post-menopausal women [23].

Lung cancer was more frequently diagnosed among men compared to women, but the relative risk of lung cancer diagnosis in women with SPN compared to those without an SPN was much higher among women compared to men, especially when the SPN was detected by chest radiograph.

These results are particularly important if we consider the clinical management of women with SPN detected during routine imaging test, especially if clinicians consider the finding less clinically significant among never-smoking patients. Moreover, these results highlight the need to include sex as a risk factor in the recommendations, as BTS have done [1], but taking into account the nodule detection pathway, CT or radiography.

Previous data have indicated that women were less likely to have immediate interventions than men after a chest radiograph [8]. This fact could be related to the perception of lung cancer as a masculine disease because of traditional higher smoking rates in men [8]. In fact, a recent systematic review and meta-analysis showed that out of the 13 randomized clinical trials carried out to evaluate the benefit of lung cancer screening, 6 (46.1%) of them included only men in the population under study [24].

We have to consider some limitations in the study. Firstly, the study uses routine clinical records and some clinical data was missing; however, there were no significant differences between the characteristics of patients with or without complete clinical information and we have dealt with these missing values as an additional category. During the follow up, electronic clinical histories were introduced in the participating hospitals and this led to improved data collection. Furthermore, by using existing data in hospital records, we were forced to limit the analysis to available data meaning that potentially relevant information (such as passive smoking) could not be included in the analysis. In addition, certain information such as smoking status was only available in the subset of patients with an SPN. In some cases, confidence intervals were large and should be interpreted with caution. Another limitation could be observer variability in the determination of the presence of SPN and its characteristics. We minimized this potential limitation by the use similar criteria for detection and description of SPN and by the assessment of the observer agreement [17].

Our aim was to evaluate the clinical practice, and thus, we included both CT and chest radiography, although the evaluations of both techniques has been challenged. Moreover, high-risk populations undergoing screening may differ quite significantly from the unselected population undergoing imaging in a routine setting (i.e., people with respiratory symptoms who seek medical care, patients with other complaints or asymptomatic patients with incidental nodules). In addition, screening trials use very strict inclusion criteria such as the inclusion of individuals aged between 50–75 years with at least a 30 pack-year tobacco habit.

## 5. Conclusions

This is the first study showing the different factors associated with SPN malignancy in men and women in routine clinical practice for both chest radiograph and CT. Factors that are traditionally associated with malignancy, such as smoking, were not significantly associated with lung cancer diagnosis or lung cancer death among women with SPN. Moreover, although the adjusted risk of lung cancer was higher among men compared to women overall, in patients with SPN the difference between men and women was not statistically significant in lung cancer diagnosis, nor mortality. All guidelines for SPN management should include factors associated with malignancy for men and women separately or introduce sex as a lung cancer predictor.

## Supporting information

S1 Table Frequency of lung cancer in to the 480 patients who underwent a chest radiograph according to their nodule’s characteristics. (DOCX)

S2 Table Lung cancer frequency according to nodule size in the patients with SPN who underwent a chest radiograph*. (DOCX)

S3 Table Frequency of lung cancer in to the 413 patients who underwent a CT according to their nodule’s characteristics. (DOCX)

S4 Table Lung cancer frequency according to nodule size in the patients with SPN who underwent a CT. (DOCX)
