# Supplementary material for: The determinants of lung cancer after detecting a solitary pulmonary nodule are different in men and women, for both chest radiograph and CT
Source: PLoS One. 2019 Sep 11;14(9):e0221134. doi: 10.1371/journal.pone.0221134 (PMC6738604; doi:10.1371/journal.pone.0221134)
Supplement: S1 Table — (DOCX) [file pone.0221134.s001.docx]

**S1 Table.** Frequency of lung cancer in to the 480 patients who underwent a chest radiograph according to their nodule’s characteristics

| **Variables N(%) (95%CI)** | | **Chest Radiography** | | | | **Men** | | | | **Women** | | | |
| --- | --- | --- | --- | --- | --- | --- | --- | --- | --- | --- | --- | --- | --- |
|  | | Total  480  (100) | No cancer  418(87.1) | Cancer  62(12.9) | p. value | Total  285 | No cancer  241 (84.6) | Cancer  44 (15.4) | p. value | Total  195 | No cancer  177 (90.8) | Cancer  18 (9.2) | p. value |
| **Diameter (mm) mean (SD)** | | 11.6 (6.0) | 10.5 (6.0) | 18.8 (7.3) | <0.001 | 12.2 (7.1) | 11 (6.3) | 19.1 (7.2) | <0.001 | 10.6(6) | 9.8(5.5) | 18.9 (6.2) | <0.001 |
| **Location** | | 0.033 | | | | 0.037 | | | |  | | | 0.557 |
|  | Upper-lobe | 263 (100) | 223 (84.8) | 40 (15.2)  (10.9-19.6) |  | 159 | 128 (80.5) | 31 (19.5)  (13.3-25.7) |  | 104 | 95 (91.4) | 9 (8.7)  (3.3-14.1) |  |
|  | Middle-lobe | 38 (100) | 38 (100) | - |  | 18 | 18 (100) | 0 |  | 20 | 20 (100) | - |  |
|  | Lower lobe | 161 (100) | 144 (89.4) | 17 (10.6)  (5.8-15.3) |  | 100 | 89 (89) | 11 (11)  (4.8-17.2) |  | 61 | 55 (90.2) | 6 (9.8)  (2.3-17.4) |  |
|  | Not available | 18 (100) | 17 (94.4) | 1 (5.6)  ((-5.3)-16.5) |  | 8 | 8 (100) | 0 |  | 10 | 9 (90) | 1 (10)  (0.1-29.7) |  |
| **Border** | | <0.001 | | | | <0.001 | | | |  | | | <0.001 |
|  | Smooth border or well defined border | 127 (100) | 122 (96.1) | 5 (3.9)  (0.5-7.3) |  | 77 | 73 (94.8) | 4 (5.2)  (0.2-10.1) |  | 50 | 49 (98) | 1 (2)  (0.1-5.9) |  |
|  | Irregular or not well define | 62 (100) | 52 (83.9) | 10 (16.1)  (6.9-25.4) |  | 37 | 30 (81.1) | 7 (18.9)  (6.1-31.7) |  | 25 | 22 (88) | 3 (12)  (0.1-25.0) |  |
|  | Lobulation | 33 (100) | 26 (78.8) | 7 (21.2)  (7-35.4) |  | 18 | 13 (72.2) | 5 (27.8)  (6.4-49.1) |  | 15 | 13 (86.7) | 2 (13.3)  (0.1-3.1) |  |
|  | Spiculation | 50 (100) | 26 (52) | 24 (48)  (34-62) |  | 40 | 22 (55) | 18 (45)  (29-3-60.6) |  | 10 | 4 (40) | 6 (60)  (27.9-92.0) |  |
|  | Not available | 208 (100) | 196 (94.2) | 12 (5.8)  (2.5-8.9) |  | 113 | 105 (92.9) | 8 (7.1)  (0.2-11.8) |  | 95 | 91 (95.8) | 4 (4.2)  (0.1-8.3) |  |
