# Supplementary material for: The determinants of lung cancer after detecting a solitary pulmonary nodule are different in men and women, for both chest radiograph and CT
Source: PLoS One. 2019 Sep 11;14(9):e0221134. doi: 10.1371/journal.pone.0221134 (PMC6738604; doi:10.1371/journal.pone.0221134)
Supplement: S2 Table — (DOCX) [file pone.0221134.s002.docx]

**S2 Table.** Lung cancer frequency according to nodule size in the patients with SPN who underwent a chest radiograph*

| **Variables N(%) (95%CI)** | **Chest Radiography** | | | | **Men** | | | | | **Women** | | |  |
| --- | --- | --- | --- | --- | --- | --- | --- | --- | --- | --- | --- | --- | --- |
|  |  | | | |  | | | | |  |  |  |  |
|  | Total  455  (100) | No cancer  396  (87) | Cancer  59  (13) | p | | Total  272 (100) | No cancer  230  (84.6) | Cancer  42 (15.4) | p | Total  183  (100) | No cancer  166 (90.4) | Cancer  17  (9.6) | p |
| **Diameter** |  |  |  | <0.001 | |  |  |  | <0.001 |  |  |  | <0.001 |
| **3-4** | 34 | 34 (100) | - |  |  | 18 | 18. (100) | - |  | 16 | 16 (100) | - |  |
| **4>8** | 134 | 131 (97.8) | 3 (2.2)  (0-4.8) |  |  | 74 | 71 (95.9) | 3 (4.1)  ((-0.4)-8.6) |  | 60 | 60 (100) | - |  |
| **8>12** | 149 | 138 (92.6) | 11 (7.4)  (3.2-11.6) |  |  | 85 | 78 (91.7) | 7 (8.3)  (2.3-14.1) |  | 64 | 60 (93.8) | 4 (6.2)  (2.5-12.2) |  |
| **12>16** | 59 | 48 (81.4) | 11 (18.6)  (8.6-28.7) |  |  | 38 | 29 (76.3) | 9 (23.7)  (10-.37.4) |  | 21 | 19 (90.5) | 2 (9.5)  ((-3.4)-22.4) |  |
| **16>20** | 21 | 14 (66.7) | 7 (33.3)  (12.7-54) |  |  | 15 | 11 (73.3) | 4 (26.7)  (3.4-49.9) |  | 6 | 3 (50) | 3 (50)  (60.1-93.9) |  |
| **20>24** | 15 | 11 (73.3) | 4 (26.7)  (3.5-61.4) |  |  | 12 | 10 (83.3) | 2 (16.7)  ((-5.4)-38.7) |  | 3 | 1 (33.3) | 2 (66.7)  (10.9-132) |  |
| **24>28** | 30 | 17 (56.7) | 13 (43.3)  (25.3-61.4) |  |  | 20 | 10 (50) | 10 (50)  (27.5-72.5) |  | 10 | 7 (70) | 3 (30)  ((-0.0-60) |  |
| **>28** | 13 | 7(53.8) | 6 (46.2)  (17.9-74.4) |  |  | 10 | 5 (50) | 5 (50)  (17.3-82.7) |  | 3 | 2 (66.7) | 1 (33.3)  ((-32.2)-98.9) |  |

*available data from 455 patients
