# Supplementary material for: The determinants of lung cancer after detecting a solitary pulmonary nodule are different in men and women, for both chest radiograph and CT
Source: PLoS One. 2019 Sep 11;14(9):e0221134. doi: 10.1371/journal.pone.0221134 (PMC6738604; doi:10.1371/journal.pone.0221134)
Supplement: S3 Table — (DOCX) [file pone.0221134.s003.docx]

**S3 Table.** Frequency of lung cancer in to the 413 patients who underwent a CT according to their nodule’s characteristics

| **Variables N(%) (95%CI)** | | **Total** | | | | **Men** | | | | **Women** | | | |
| --- | --- | --- | --- | --- | --- | --- | --- | --- | --- | --- | --- | --- | --- |
|  | | **Total**  413 (100) | **No cancer**  336 (81.4) | **Cancer**  77 (18.6) | **p.value** | **Total**  261  (100) | **No cancer**  202  (77.4) | **Cancer**  59  (22.6) | **p.value** | **Total**  152 (100) | **No cancer**  134 (88.2) | **Cancer**  18 (11.8) | **p.value** |
| **Diameter (mm) mean (SD)** | | 10.5 (6.8) | 8.9 (5.6) | 17 (7.7) | <0.001 | 12.2 | 8.9 (5.3) | 19.1 (7.2) | <0.001 | 10.6 (6.4) | 8.8 (6.0) | 12 .7 (7.1) | 0.02 |
| **Localization** | | 0.266 | | | | 0.125 | | | | 0.966 | | | |
|  | Upper-lobe | 200 (100) | 157  (78.5) | 43 (21.5) (15.8-27.2) |  | 137  (100) | 101 (73.7) | 36 (26.3)  (18.9-33.7) |  | 63  (100) | 56 (88.9) | 7 (11.1)  (3.3-18.9) |  |
|  | Middle-lobe | 50  (100) | 44  (88) | 6 (12)  (2.9-21.1) |  | 26  (100) | 23 (88.5) | 3 (11.5)  ((-1)-24.1) |  | 24  (100) | 21 (87.5) | 3 (12.5)  ((-0.1)-2.6) |  |
|  | Lower lobe | 150 (100) | 125 (83.3) | 25 (16.7)  (10.7-22.7) |  | 91  (100) | 74 (81.3) | 17(18.7)  (10-6-26.8) |  | 59  (100) | 51 (86.4) | 8 (13.6)  (4.7-22.4) |  |
|  | Not available | 13  (100) | 12 (92.3) | 1 (7.7)  ((-0.7)-22.8) |  | 7  (100) | 7 (100) | 0 |  | 6  (100) | 5 (83.3) | 1 (16.7)  (0.1-49.4) |  |
| **Border** | | <0.001 | | | | <0.001 | | | |  | | | 0.001 |
|  | Smooth border or well defined border | 88  (100) | 85 (96.6) | 3 (3.4)  ((-0.4-7.2) |  | 50  (100) | 48 (96) | 3 (4)  (0.)-10.2) |  | 38  (100) | 37 (97.4) | 1 (2.6)  (0.1-9.2) |  |
|  | Irregular or not well define | 44  (100) | 37 (84.1) | 7 (15.9)  (4.9-26.9) |  | 28  (100) | 24 (85.7) | 4 (14.3)  (1.2-27.5) |  | 16  (100) | 13 (81.3) | 3 (18.8)  (0.1-38.5) |  |
|  | Lobulation | 41  (100) | 25 (61) | 16 (39)  (23.9-54.2) |  | 23  (100) | 13 (56.5) | 10(43.5)  (22.7-64.2) |  | 18  (100) | 12 (66.7) | 6 (33.3)  (10.8-55.8) |  |
|  | Spiculation | 67  (100) | 35 (52.2) | 32 (47.8) (35.7-59.8) |  | 56  (100) | 28 (50) | 28 (50)  (36.7-63.2) |  | 11  (100) | 7 (63.6) | 4 (36.4)  (6.4-66.3) |  |
|  | Not available | 173 (100) | 156 (90.2) | 17 (9.8) (5.4-14.3) |  | 104  (100) | 92 (88.5) | 12 (11.5)  (5.4-17.7) |  | 69  (100) | 64 (92.8) | 5 (7.2)  (1-13.4) |  |
| **Opacity** | | | | | | | | | | | | | |
|  | Solid | 225 (100) | 190  (84.4) | 35 (15.6) (9.2-15) | 0.087 | 143  (100) | 118 (82.5) | 25 (17.5)  (11.2-23.7) | 0.080 | 82  (100) | 72 (87,8) | 10 (12.2)  (5-19.3) | 0.642 |
|  | Ground glass | 14  (100) | 11  (78.6) | 3 (21.4)  (0-43.8) |  | 8  (100) | 5 (62.5) | 3 (37.5)  (15.5-73.4) |  | 6  (100) | 6 (100) | 0 |  |
|  | Partly solid | 16  (100) | 16 (78.6) | - |  | 15  (100) | 11 (73.3) | 4 (26.7)  (34.5-49.9) |  | 10 (100) | 10 (100) | 0 (0) |  |
|  | Calcification | 25  (100) | 21 (84) | 4 (16)  (1.3-30.7) |  | 11  (100) | 11 (100) | 0 |  | 5  (100) | 5 (100) | 0 (0) |  |
|  | Not available | 133 (100) | 100 (75.2) | 33 (24.8)  (17.4-32.2) |  | 84  (100) | 60 (71.4) | 24 (28.6)  (18.9-38.3) |  | 49  (100) | 40 (81.6) | 9 (18.4)  (7.4-29.4) |  |
