# Supplementary material for: The determinants of lung cancer after detecting a solitary pulmonary nodule are different in men and women, for both chest radiograph and CT
Source: PLoS One. 2019 Sep 11;14(9):e0221134. doi: 10.1371/journal.pone.0221134 (PMC6738604; doi:10.1371/journal.pone.0221134)
Supplement: S4 Table — (DOCX) [file pone.0221134.s004.docx]

**S4 Table.** Lung cancer frequency according to nodule size in the patients with SPN who underwent a CT*

|  | | **Total** | | | |  | | **Men** | | | | | **Women** | | | | |
| --- | --- | --- | --- | --- | --- | --- | --- | --- | --- | --- | --- | --- | --- | --- | --- | --- | --- |
| **Variables N(%) (95%CI)** | | Total  395  (100) | No cancer  335  (84.8) | Cancer  60  (1.5) | p | | Total  251 | | No cancer  193  (76.9) | Cancer  58  (23.1) | P | Total  146 | | No cáncer  131  (87.9) | Cancer  18  (12.1) | p |  |
| **Diammeter (mm)** | |  |  |  | <0.001 | |  | |  |  | <0.001 |  | |  |  | 0.037 |  |
|  | 3-4 | 47 | 45(97.8) | 2 (4.3)  (0.1-10.1) |  |  | 23 | | 22 (95.6) | 1 (4.4)  (0.1-12.9) |  | 24 | | 23 (95.8) | 1 (4.2)  (0.1-12.4) |  |  |
|  | 4>8 | 165 | 153 (92.8) | 12 (7.3)  (3.3-11.3) |  |  | 100 | | 95 (95) | 5 (5)  (0.6-9.3) |  | 65 | | 58 (89.2) | 7 (10.8)  (3.1-18.4) |  |  |
|  | 8>12 | 76 | 67 (86.2) | 9 (11.8)  (4.5-19.2) |  |  | 48 | | 42 (87.5) | 6 (12.5)  (3-22) |  | 28 | | 25 (85.3) | 3 (10.8)  (0.1-22.4) |  |  |
|  | 12>16 | 41 | 31 (75.6) | 10 (24.4)  (11.1-37.7) |  |  | 25 | | 18 (72) | 7 (28)  (9.9-46) |  | 16 | | 13 (81.3) | 3 (18.7)  (0.1-38.6) |  |  |
|  | 16>20 | 18 | 16 (57.1) | 12 (42.9)  (24.2-6.5) |  |  | 21 | | 11 (52.4) | 10 (47.6)  (25.7-69.6) |  | 7 | | 5 (71.4) | 2 (28.6)  (0.1-65.8) |  |  |
|  | 20>24 | 16 | 5 (31.3) | 11 (75)  (53.1-96.9) |  |  | 13 | | 3 (23.1) | 10 (76.9)  (53-100) |  | 3 | | 1 (33.3) | 2  (66.7)  (1.9)-132.3) |  |  |
|  | 24>28 | 19 | 5 (26.3) | 14 (73.7)  (53.3-94.1) |  |  | 17 | | 4 (23.5) | 13 (76.5)  (55.6-97.3) |  | 2 | | 1 (50) | 1 (50)  (0.1-148) |  |  |
|  | >28 | 8 | 5 (62.5) | 3 (37.5)  (15.9-73.4) |  |  | 4 | | 1 (25) | 3 (75)  (25.9-124) |  | 4 | | 4 (100) | 0 |  |  |

*available data from 395 patients
